# Association between adverse pregnancy outcome and imbalance in angiogenic regulators and oxidative stress biomarkers in gestational hypertension and preeclampsia

**DOI:** 10.1186/s12884-015-0624-y

**Published:** 2015-08-25

**Authors:** Cornelius A. Turpin, Samuel A. Sakyi, William KBA Owiredu, Richard KD. Ephraim, Enoch O. Anto

**Affiliations:** Department of Molecular Medicine, School of Medical Sciences, Kwame Nkrumah University of Science and Technology (KNUST), Kumasi, Ghana; Department of Obstretics & Gynaecology, Komfo Anokye Teaching Hospital, Kumasi, Ghana; Department of Bacteriology, Noguchi Memorial Institute for Medical Research, Accra, Ghana; Department of Medical Laboratory Technology, University of Cape Coast, Cape Coast, Ghana

## Abstract

**Background:**

Gestational hypertension (GH) and Preeclampsia, (PE) are the most complicated amongst hypertensive disorders of pregnancy. The mechanism that links hypertension in pregnancy to adverse maternal outcomes is not fully understood though some relate this to endothelial dysfunction originating from an imbalanced angiogenic regulators and oxidative stress biomarkers. This study assessed the correlation between angiogenic regulators and oxidative stress biomarker levels with adverse pregnancy outcomes among GH and PE participants.

**Methods:**

A cohort of pregnant women who received antenatal care at the Obstetrics and Gynaecology department of the Komfo Anokye Teaching Hospital (KATH) were followed. During their antenatal visits, 100 developed PE and 70 developed GE, of these, 50 PE and 50 GH gave informed consent. Their blood samples were taken at time of diagnosis and 48 h post-partum. 50 other aged-matched women who did not develop neither GH nor PE were selected as controls. Placental growth factor (PLGF), soluble fms-like tyrosine kinase 1 (sFlt-1) and 8-epi-prostaglandin F2alpha (8-epi-PGF2α) levels were estimated by ELISA and total antioxidant capacity (T-AOC) was measured spectrophotometrically. Graphpad Prism was used for data analysis.

**Results:**

Median levels of sFlt-1, 8-epi-PGF2α and sFlt-1/PLGF were elevated among participants with PE co-existing with intrauterine fetal death (IUFD), placental abruptio, placental previa, HELLP syndrome and intrauterine growth restriction (IUGR) compared to PE without adverse outcomes (*p* = 0.041, *p* = 0.005, *p* = 0.0002). Levels of PLGF, T-AOC and PLGF/sFlt-1 were significantly reduced among participants with PE co-existing with IUFD, placental abruptio, placental previa, HELLP syndrome and IUGR compared to PE without adverse outcomes (*p* = 0.0013, *p* = 0.006, *p* < 0.0001). A significant negative correlation of IUGR (*p* = 0.0030; *p* < 0.0001), placental abruptio (*p* < 0.0001; *p* < 0.0001), IUFD (*p* < 0.0001; *p* < 0.0001), stillbirth (*p* = 0.0183 and *p* < 0.000), and postpartum haemorrhage (PPH) (*p* = 0.0420; *p* = 0.0044) were associated with both PLGF and T-AOC whilst a significant positive correlation of IUGR, placental abruptio (*p* < 0.0001; *p* < 0.0001), IUFD (*p* < 0.0001; *p* < 0.0001), stillbirth (*p* < 0.0001; *p* < 0.0001), and PPH (*p* = 0.0043; *p* = 0.0039) were observed with both sFlt-1 and 8-epi-PGF2α in PE.

**Conclusions:**

Imbalance in the levels of angiogenic regulators and oxidative stress biomarkers correlates with adverse pregnancy outcomes among PE participants. Early identification of these imbalance would alert health care givers in anticipation of adverse pregnancy outcome and thus increased surveillance during pregnancy and parturition and measures to ameliorate the adverse outcome.

## Background

Preeclampsia, the most complicated amongst hypertensive disorders of pregnancy is enigmatic due to its multisystemic nature and associated adverse pregnancy outcomes [1]. The mechanism of how some pregnancies lead to adverse maternal outcome is not fully understood though endothelial dysfunction, advanced maternal age and gestational hypertension has been suggested to play a role [2, 3]. Adverse pregnancy outcome is any event which reduces the chance of having a healthy baby and includes: stillbirth, preterm delivery, foetal anomalies, intrauterine growth retardation (IUGR), intrauterine fetal death (IUFD) and small for gestational age (SGA) infants. These adverse outcomes have been linked with preeclampsia [4]. Despite this link, little or not much research have explored the association between both antepartum and postpartum adverse pregnancy outcomes, and angiogenic regulators and oxidative stress biomarker levels. Placental released proteins such as soluble fms-like tyrosine kinase-1 (sFlt1) and placental growth factor (PLGF) are angiogenic regulators which levels varies considerably in circulation during pregnancy [5]. A previous published study found an increased levels of sFlt-1 and a decreased PLGF concentrations to be associated with IUGR, spontaneous preterm birth and stillbirth [6] whilst other study found an inconsistent result [7]. Pregnancies complicated by low birth weight (LBW), spontaneous preterm delivery, and small for gestational age (SGA) infants have been shown to have reduced antioxidant and increased pro-oxidant levels, although no correlational data have been explored to establish this link [8]. Ghana’s attempt to achieve the millennium development goals (MDG) four and five has not been successful due to high incidence of adverse pregnancy outcomes. The association between angiogenic regulators and oxidative stress levels may share a common etiological pathway, moreover, correlational data between angiogenic regulators, oxidative stress biomarker levels and adverse pregnancy outcome in PE have not been published in any previous studies. It is against this background that this study seek to assess the correlation between angiogenic regulators and oxidative stress biomarker levels with adverse outcomes in gestational and preeclamptic women.

## Methods

### Study design and study setting

This hospital based prospective cohort study was carried out from April to November, 2014 at the Obstetrics and Gynaecology (O & G) department of KATH in Kumasi. Kumasi is in the Ashanti Region of Ghana and has an average population of 4,780,380 (Ghana Statistical service, 2012). KATH is the second largest tertiary hospital in Ghana with a thousand (1000) bed capacity. It serves as a major referral centre for the middle belt and northern part of Ghana. The hospital also receives referrals from other regions and this gives fair representation of the Ghanaians population.

### Selection of participants

A cohort of pregnant women who patronize antenatal services at the O&G department of KATH were followed. During their periodic visits, 100 developed PE, and 70 developed GH. Some were lost to follow ups and some refused to give an informed consent. Finally, 50 GH, and 50 PE pregnant women gave written informed consent and their blood samples were taken at the time of diagnosis and 48 h postpartum. 50 other aged-matched pregnant women who did not develop neither GH nor PE were used as normal controls. The diagnosis of hypertensive disorders of pregnancy was done by qualified Obstetrician/Gynaecologist using the National High Blood Pressure Education Program Working Group diagnostic criteria (NBPEPWG, 2000). Information relating to obstetric and demographic characteristics were obtained from record reviews of hospital database and structured closed ended questionnaires. Preeclampsia was defined as the onset, after 20 weeks of gestation for both hypertension (>140/90 mmHg) and dipstick proteinuria (> + 0.3 g/l). Gestational hypertension was defined as hypertension occurring after 20 weeks of gestation, without dipstick proteinuria [9].

### Inclusion criteria and exclusion criteria

Nulliparous and multiparous pregnant women aged 18–40 years, within the gestational age of ≥20–40 weeks with singleton pregnancy were included in this study. Participants previously diagnosed with chronic hypertension, heart disease, diabetes mellitus, renal disease or are on antihypertensive therapy as well as those who were unable to give informed consent were excluded from the study.

### BP measurements

Trained personnel used a mercury sphygmomanometer (Accoson, England) and a stethoscope (3M™ Littmann® Stethoscopes, USA) to measure the blood pressure of participants in accordance with recommendations of the American Heart Association [10]. The procedure was repeated for each patient between 5 and10 min. Mean values of duplicate measurements were recorded as the blood pressure to the nearest 2.0 mmHg.

### Urine sample collection and estimation of proteinuria

Participants provided 10–20 ml freshly voided early morning urine in clean, wide mouth and leak proof containers. Semi quantitative proteinuria was immediately assessed using dipstick (URIT 2V^PG^ Medical electronic Co., Ltd. China). Proteinuria was defined as the presence of urinary protein in concentrations more than 0.3 g/l or ≥ + using the semi-quantitative colour scale on the urine reagent dipstick on urine dipstick [9].

### Ethical consideration

Ethical approval was granted by the Committee on Human Research, Publications and Ethics (CHRPE) (CHRPE/AP/365/14), School of Medical Sciences, Kwame Nkrumah University of Science & Technology (KNUST) and the Research and Development Unit of the Komfo Anokye Teaching Hospital (KATH). Patients who gave informed written consent were involved in the study. All data were de-identified before analysis.

### Blood sample collection

10mls of venous blood sample was collected from each participants. Blood was dispensed into serum separator tubes and centrifuged (Nüve NF 200, Germany) at 7000 rpm for 15 min. Serum was aliquoted under sterile conditions and stored at −80 °C (Thermo Scientific™ Revco™ UxF − Ultra-Low Temperature Freezers, USA) until assay.

### Measurement of sFlt-1, PLGF and 8-epi-PGF2α

Serum levels of sFlt-1, PLGF and 8-epi-PGF2α were measured in duplicate using commercially available ELISA kits from R&D System Inc. (Minneapolis, MN USA). The optical density was measured at 450 nm using microplate ELISA reader (Mindray MR-96A). The plasma levels of each factor were calculated using standard curves derived from a known concentration of the respective recombinant factors.

### Total antioxidant capacity (T-AOC) assay

Total antioxidant capacity (TAOC) reagents was obtained from Green stone Swiss Co., Ltd, China and serum levels were estimated spectrophotometrically (Mindray BA-88A, China) at 593 nm. This assay was measured based on the ferric reducing ability of plasma (FRAP) method. The measurement of the ferric reducing ability of plasma (FRAP) was done by the assay protocol as described by Benzie and Strain, (1999). All samples were analyzed in triplicate.

### Definition of obstetric terms

IUGR was defined as poor growth of a fetus while in the mother’s womb during pregnancy [11]. Severe preeclampsia was defined as a systolic blood pressure (SBP) ≥160 mmHg or a diastolic blood pressure (DBP) ≥110 mmHg on two occasions recorded 6-h apart in association with proteinuria (≥ +3) [12]. Preterm delivery was defined as delivery before 37 completed weeks of gestation [13]. Stillbirth was defined based on International Classification of Diseases, 10th revision (ICD-10) as “death prior to the complete expulsion or extraction from its mother of a product of conception, with respective to the duration of pregnancy [14]. IUFD was defined as the fetal death (where there is no sign of life) at equal or more than 20 weeks of gestation and/or birth weight of equal or more than 500 gram [15]. An abruptio placenta was defined as premature separation of normally placed placenta from uterine wall after 20 weeks of gestation and prior to birth [16]. Placenta previa was defined as implantation of the placenta over or near the internal orifices of the cervix after 20 weeks gestation prior to transvaginal or abdominal ultrasonography [17]. Premature pre-rupture of membranes (PPROM) was defined as rupture of the membrane of the amniotic sac and chorion occurring before the onset of labour [18].

### Statistical analysis

Statistical analysis was performed using software Graphpad Prism, version 5.0 (Graph Pad Software Inc., Los Angeles) for windows. Comparison between contingency variables was performed using Chi-square test. Data were reported as mean ± standard deviation (SD) for continuous data, as median (interquartile range) for non-parametric parameters (biomarkers) and as a frequency (percentage) for categorical data. The correlations between angiogenic factors and oxidative stress and adverse outcome were determined by Spearman correlation analysis. Statistical significance was accepted at *p* < 0.05 for all comparisons.

## Results

### Socio-demographic, obstetric and clinical characteristic

The mean age of the general study participant was 29.78 years. Greater proportion (83.3 %) of the pregnant women were married whilst 16.7 % were singles. The percentage of married participants with PE (80.0 %) and GH (76.0 %) were significantly lower compared to the normal pregnant women (94.0 %) (*p* = 0.0173). Among the 55.3 % of the participant who had completed primary education, 72.0 % developed PE and 58.0 % had GH compared to 36.0 % among normal pregnant women (*p* = 0.0052). A higher percentage (42.7 %) of the participants were nulliparous whilst 35.3 and 22.0 % were multiparous and primiparous respectively. Majority of the participants were multigravida 59/150 (39.3 %) of which 52.0 % developed GH and 24.0 % had PE. Significantly higher proportion of preeclamptic participants than GH and NP had spontaneous abortion (64.0 % vs 44.0 % vs 42.0 %; *p* = 0.0288), family history of hypertension (34.0 % vs 8.0 % vs 2.0; *p* < 0.0001) and previous caesarean section (48.0 % vs 18.0 % vs 12.0 %; *p* < 0.0001). Participants with PE and GH had a significantly higher mean levels of systolic blood pressure (SBP) (*p* = 0.0020), diastolic blood pressure (DBP) (*p* = 0.0079) and proteinuria (*p* < 0.0001) compared to normotensive pregnant women (*p* < 0.05) (Table 1).

**Table 1:** Sociodemographic, obstetric and clinical characteristics of study Participants

Variables	Total (*n* = 150)	NP (*n* = 50)	GH (*n* = 50)	PE (*n* = 50)	*p-value*
Mean age (years)	29.8 ± 0.4	30.79 ± 0.7	30.49 ± 0.8	28.85 ± 0.6	0.8992
Marital status					0.0179
Single	25 (16.7 %)	3 (6.0 %)	12 (24.0 %)	10 (20.0 %)	
Married	125 (83.3 %)	47 (94.0 %)	38 (76.0 %)	40 (80.0 %)	
Level of education					0.0052
No education	5 (3.3 %)	0 (0.0 %)	2 (4.0 %)	3 (6.0 %)	
Primary	83 (55.3 %)	18 (36.0 %)	29 (58.0 %)	36 (72.0 %)	
Secondary	37 (24.7 %)	12 (24.0 %)	19 (38.0 %)	6 (12.0 %)	
Tertiary	28 (18.6 %)	20 (40.0 %)	3 (6.0 %)	5 (10.0 %)	
GA at baseline	24.1 ± 4.0	24.3 ± 5.1	23.81 ± 4.4	24.39 ± 2.6	0.9102
GA at delivery	37.0 ± 0.7	38.55 ± 0.4	36.91 ± 0.4	35.62 ± 0.3	0.5711
Parity					0.7601
nulliparous	64 (42.7 %)	20 (40.0 %)	19 (38.0 %)	25 (50.0 %)	
primiparous	33 (22.0 %)	12 (24.0 %)	11 (22.0 %)	10 (20.0 %)	
multiparous	53 (35.3 %)	18 (36.0 %)	20 (40.0 %)	15 (30.0 %)	
Gravidity					0.0501
primigravida	44 (29.3 %)	12 (24.0 %)	14 (28.0 %)	18 (36.0 %)	
Secundigravida	47 (31.3 %)	17 (34.0 %)	10 (20.0 %)	20 (40.0 %)	
multigravida	59 (39.3 %)	21 (42.0 %)	26 (52.0 %)	12 (24.0 %)	
Family history of HTN					
Yes	22 (14.7 %)	1 (2.0 %)	4 (8.0 %)	17 (34.0 %)	<0.0001
History of abortion					
Yes(spontaneous)	75 (50.0 %)	21 (42.0 %)	22 (44.0 %)	32 (64.0 %)	0.0288
Previous caesarean section					
Yes	39 (26.0 %)	6 (12.0 %)	9 (18.0 %)	24 (48.0 %)	<0.0001
BP (mmHg)					
SBP	146.1 ± 1.5	114.3 ± 1.0	158.8 ± 1.9^b^	165.2 ± 1.6^b^	0.0020
DBP	93.8 ± 1.1	69.33 ± 1.0	103.4 ± 1.2^b^	108.6 ± 1.1^b^	0.0079
Urinary protein (g/l)	0.7 ± 0.1	0.01 ± 0.0	0.150 ± 0.0^a^	2.05 ± 0.1^a^	<0.0001

Values are presented as frequency (proportion) and mean ± SD. *p* < 0.05 is considered statistically significant difference. ^a^significant compared to NP; ^b^significant compared to NP

### Baseline and postpartum levels of angiogenic regulators and oxidative stress biomarkers

Median levels of sFlt-1, 8-epi-PGF2α and sFlt-1/PLGF were significantly elevated in PE > GH > NP (*p* < 0.0001) whilst levels of PLGF, T-AOC and PLGF/sFlt-1 were significantly reduced in PE > GH > NP (*p* < 0.0001) before delivery. Median levels of PLGF, PLGF/sFlt-1 ratio and T-AOC increased in PE, GH and NP at 48 h after delivery compared levels before delivery (*p* < 0.0001) whilst levels of sFlt-1, sFlt-1/PLGF ratio and 8-epi-PGF2α decreased at 48 h after delivery in PE, GH, and NP compared to levels before delivery (*p* < 0.0001) (Table 2).

**Table 2:** Baseline and Postpartum levels of Angiogenic and oxidative biomarkers among the studied groups

Parameters	NP		GH		PE	
	Pre-delivery	48 h postpartum	Pre-delivery	48 h postpartum	Pre-delivery	48 h postpartum
Angiogenic Factors						
PLGF (pg/ml)	137.7	187.9**	53.05	163.9***	21.40	96.3***
	(88.1–177.8)	(130.7–214.6)	(38.33–74.80)	(94.00–200.3)	(12.40–48.40)	(77.3–131.0)
sFlt-1 (pg/ml)	119.5	45.6***	413.2	77.3***	787.6	104.6***
	(87.4–160.4)	(19.28–88.7)	(164.5–635.0)	(44.4–101.6)	(510.7–926.3)	(66.3–139.0)
sFlt-1/PLGF	0.9	0.2***	7.8	0.5***	36.80	1.1***
	(0.6–1.2)	(0.2–0.5)	(5.4–12.8)	(0.2–0.7)	(16.42–56.92)	(0.3–2.0)
PLGF/sFlt-1	1.1	3.4**	0.1	2.2***	0.0351	0.9***
	(0.5–2.0)	(1.9–9.5)	(0.1–0.5)	(1.0–3.7)	(0.015–0.085)	(0.5–1.7)
Oxidative biomarkers						
8-epi-PGF2α (pg/ml)	35.7	15.8**	198.8	18.4***	324.6	66.3***
	(28.7–42.9)	(12.2–18.7)	(92.48–392.3)	(12.0–32.1)	(194.2–618.6)	(32.65–100.4)
TAOC (mmol/l)	1.1	1.2*	0.6	0.8*	0.5	0.7***
	(1.0–1.2)	(1.1–1.3)	(0.4–0.8)	(0.6–1.1)	(0.2–0.6)	(0.4–0.9)

Values are presented as median (Interquartile range). *p < 0.05; **p < 0.001; ***p < 0.0001

### Antepartum and postparturm obstetric outcomes

Table 3 shows antepartum and postpartum obstetric complications and outcomes among study participants. A greater proportion of study participants had cephalic presentation. A significant higher proportion of preeclamptics (24.0 %) had breech presentation compared to NP (0.0 %) and GH (8.0 %). There were increased proportion of placental praevia (14.0 % vs 0.0 %), placental abruptio (22.0 % vs 0.0 %), IUFD (40.0 % vs 0.0 %), IUGR (44.0 % vs 1.0 %), APH (22.0 % vs 0.0 %) and PPROM (16.0 % vs 0.0 %) in PE compared to NP control (*p* < 0.05). Significantly, higher proportion (86.0 %) of PE and 28.0 % of GH were delivered by emergency caesarean section compared to NP controls (0.0 %). Presentations such as fresh stillbirth (24.0 % vs 2.0 %), PPH (26.0 % vs 6.0 %), preterm delivery (90.0 % vs 6.0 %) and maternal mortality (10.0 % vs 0.0 %) were significantly associated with PE pregnancies compared to NP controls. However, prolonged obstructed labour was mostly common in GH pregnancies compared NP pregnancies (58 % vs 10.0 %).

**Table 3:** 0-Antepartum and Postpartum obstetric characteristic and adverse complications

Variables	NP(*n* = 50)	GH(*n* = 50)	PE(*n* = 50)
Fetal presentation			
Cephalic/Vetex	50(100.0 %)	46(92.0 %)	38(76.0 %)
breech	0(0.0 %)	4(8.0 %)	12(24.0 %)
Placental praevia			
Yes	0(0.0 %)	4(8.0 %)	7(14.0 %)
Placental abruption			
Yes	0(0.0 %)	1(2.0 %)	11(22.0 %)
IUFD			
Yes	0(0.0 %)	5(10.0 %)	20(40.0 %)
IUGR			
Yes	1(2.0 %)	7(14.0 %)	22(44.0 %)
APH			
Yes	0(0.0 %)	2(8.0 %)	11(22.0 %)
PPROM			
Yes	0(0.0 %)	0(0.0 %)	8(16.0 %)
Mode of delivery			
*Vaginal*			
Spontaneous	45(90.0 %)	15(30.0 %)	0(0.0 %)
Induced	5(10.0 %)	20(40.0 %)	1(2.0 %)
*Caesarean section*			
Emergency	0(0.0 %)	14(28.0 %)	43(86.0 %)
Elective	0(0.0 %)	1(2.0 %)	7(14.0 %)
Stillbirth			
Fresh	1(2.0 %)	5(10.0 %)	12(24.0 %)
Macerated	0(0.0 %)	1(2.0 %)	2(4.0 %)
Live birth			
Yes	49(98.0 %)	44(88.0 %)	36(72.0 %)
Prolong labour			
Yes	5(10.0 %)	29(58.0 %)	13(26.0 %)
PPH			
Yes	3(6.0 %)	5(10.0 %)	13(26.0 %)
Status of delivery			
Term	47(94.0 %)	12(24.0 %)	5(10.0 %)
preterm	3(6.0 %)	38(76.0 %)	45(90.0 %)
Maternal mortality			
Yes	0(0.0 %)	0(0.0 %)	5(10.0 %)

Values are presented as frequency (proportion) unless otherwise indicated

### Angiogenic and oxidative stress levels categorized to severity of the condition

**Fig. 1:**
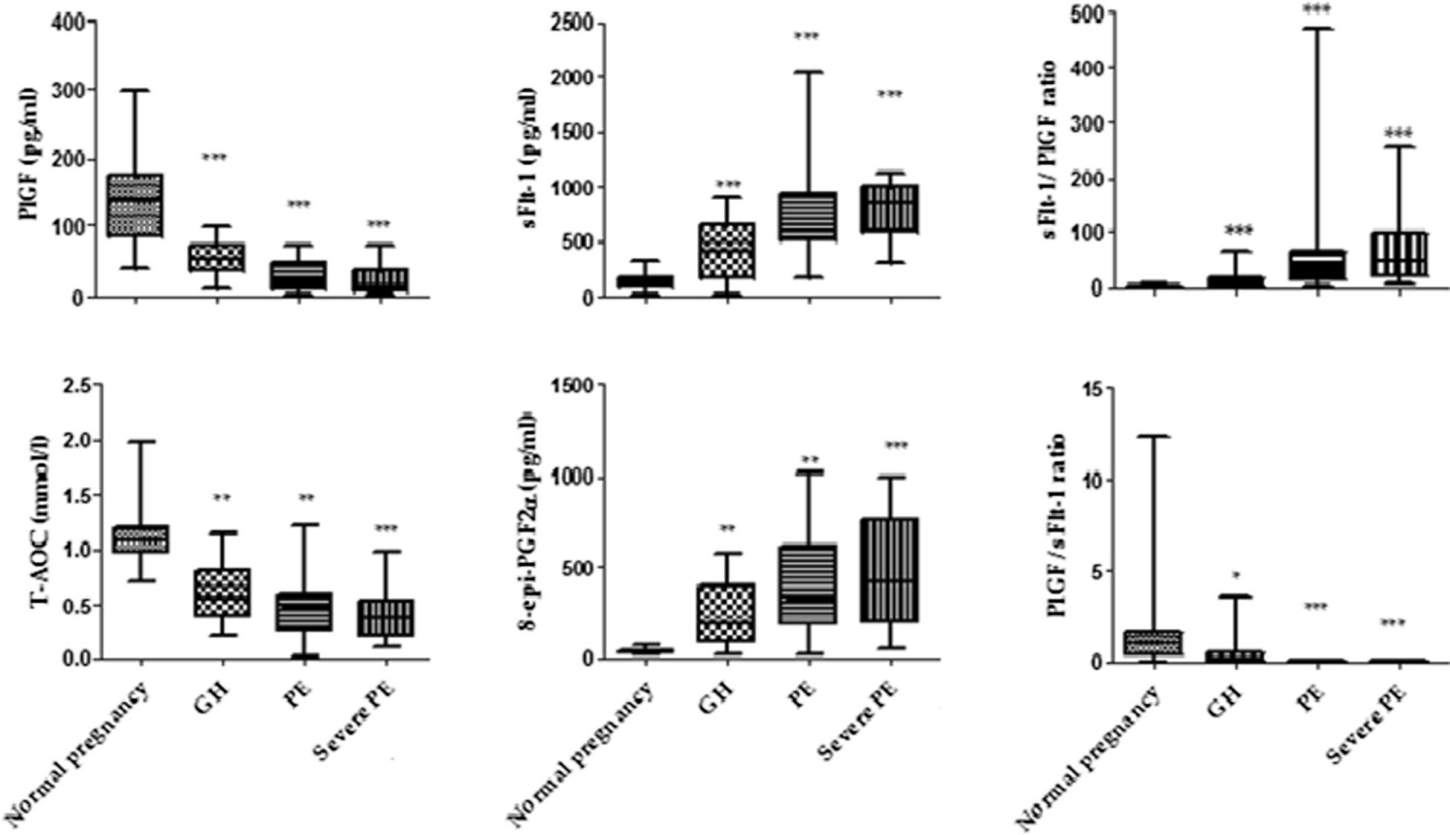
Median levels of angiogenic and oxidative stress biomarkers stratified by Hypertensive disorders

Figure 1 summarizes the levels of angiogenic and oxidative stress markers in relation to severity of pregnancy condition. Median levels of PLGF, PLGF/sFlt-1 and T-AOC decreased in the order of severity (severe PE < PE < GH < NP; *p* < 0.0001) whilst sFlt-1, 8-epi-PGF2α and sFlt- 1/PLGF ratio significantly increased in study participants in the order of severity (severe PE > PE > GH > NP; *p* < 0.0001) (Fig. 1).

### Angiogenic and oxidative stress levels in relation to adverse pregnancy outcomes in PE

**Fig. 2:**
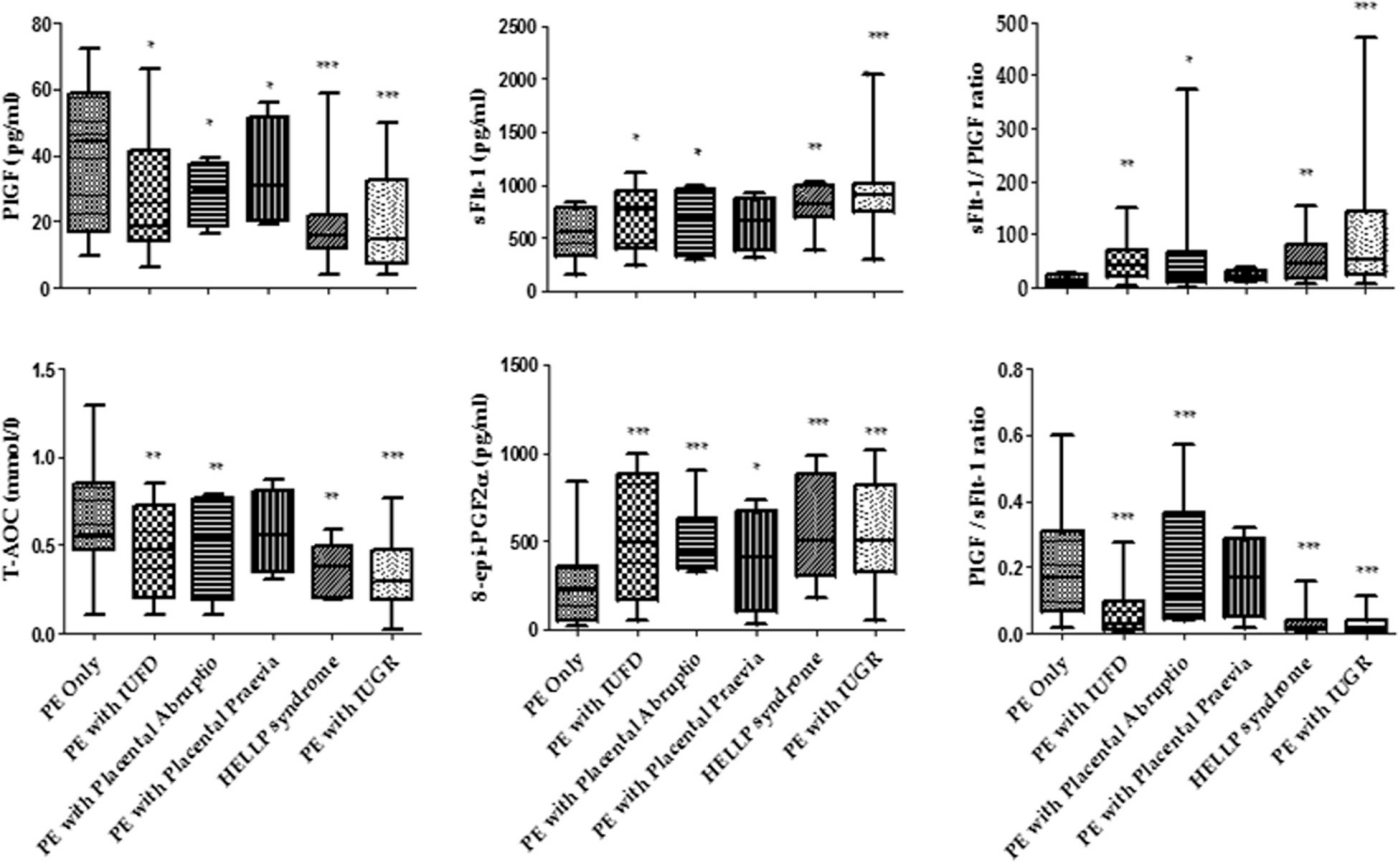
Levels of angiogenic and oxidative stress biomarkers in PE-co-related adverse outcomes

Figure 2 shows the levels of angiogenic and oxidative stress markers in PE and PE co-associated adverse outcome. Median Levels of sFlt-1, 8-epi-PGF2α and sFlt-1/PLGF were significantly elevated among study participants with PE co-existing with IUFD, placental abruptio, placental previa, HELLP syndrome and IUGR compared to PE without adverse outcome (*p* = 0.041, *p* = 0.005, *p* = 0.0002). Levels of PLGF, T-AOC and PLGF/sFlt-1 were significantly reduced in study participants with PE co-existing with IUFD, placental abruptio, placental previa, HELLP syndrome and IUGR compared to PE without adverse outcome (*p* = 0.0013, *p* = 0.006, *p* < 0.0001 respectively) (Fig. 2).

### Birthweight of babies according to studied groups

**Fig. 3:**
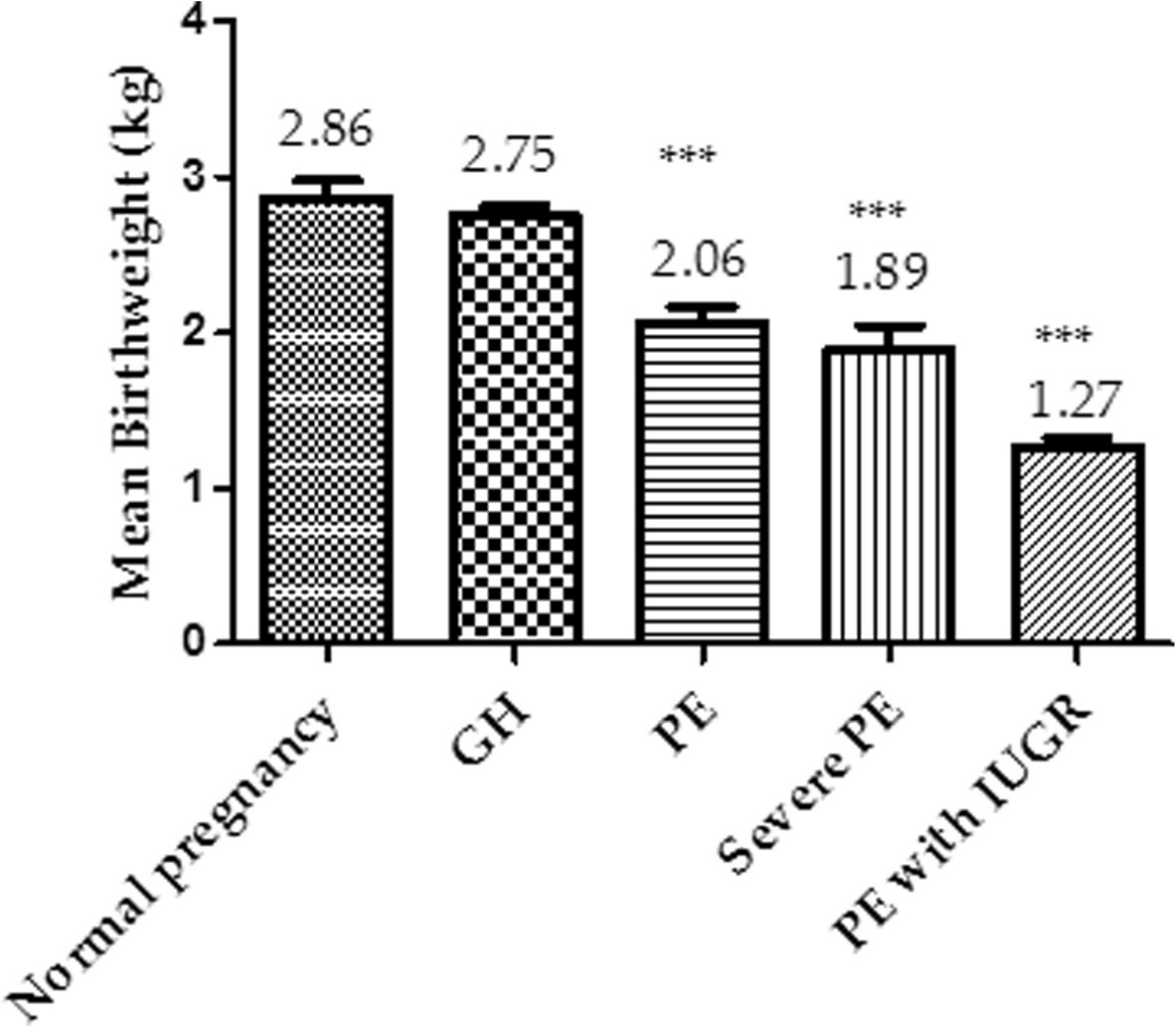
Birthweight of babies stratified by the severity of the condition

As shown in Fig. 3, babies of pregnant women with PE associated IUGR had significantly low birthweight (LBW) compared to those with only PE, GH, and NP (*p* < 0.0001) (Fig. 3).

### Correlation of angiogenic and oxidative biomarkers with antepartum and postpartum adverse pregnancy outcome in PE

Table 4 shows Spearman rho correlation of angiogenic and oxidative stress biomarkers with antepartum and adverse maternal outcomes in preeclamptic pregnancy. There was a significant (*p* < 0.05) negative correlation of PLGF and T-AOC with IUGR, placental abruptio, IUFD, stillbirth, and PPH. Conversely, there was a significant positive correlation (*p* < 0.05) of sFlt-1 and 8-epi-PGF2α with IUGR, placental abruptio, IUFD, stillbirth, and PPH (Table 4).

**Table 4:** Spearman rho correlation of Angiogenic and Oxidative biomarkers with antepartum and postpartum adverse pregnancy outcome in Preeclampsia women

	PLGF	sFlt-1	8-epi-PGF2α	T-AOC
Antepartum complications				
IUGR	*r* = −0.416;	*r* = 0.549;	*r* = 0.610;	*r* = −0.635;
	*p* = 0.0030	*p* < 0.0001	*p* < 0.0001	*p* < 0.0001
Placental abruptio/praevia	*r* = −0.663;	*r* = 0.680;	*r* = 0.579;	*r* = −0.740;
	*p* < 0.0001	*p* < 0.0001	*p* < 0.0001	*p* < 0.0001
IUFD	*r* = −0.694;	*r* = 0.730;	*r* = 0.629;	*r* = −0.819;
	*p* < 0.0001	*p* < 0.0001	*p* < 0.0001	*p* < 0.0001
Postpartum adverse outcomes				
Stillbirth	*r* = −0.366;	*r* = 0.732;	*r* = 0.691;	*r* = −0.815;
	*p* = 0.0183	*p* < 0.0001	*p* < 0.0001	*p* < 0.0001
PPH	*r* = −0.297;	*r* = 0.479;	*r* = 0.410;	*r* = −0.509;
	*p* = 0.0420	*p* = 0.0043	*p* = 0.0039	*p* = 0.0044

r: correlation coefficient; *p* < 0.05 (statistically significant) *p* < 0.001 (statistically highly significant) *p* < 0.0001 (statistically very highly significant). *r* < 0.5 (weak correlation); *r* > 0.5 (strong correlation)

### Correlation of angiogenic and oxidative biomarkers with antepartum and postpartum adverse pregnancy outcome in GH

Table 5 shows Spearman rho correlation of angiogenic and oxidative stress biomarkers with antepartum and adverse maternal outcomes in GH. In general there were no significant correlation between angiogenic factor, oxidative stress biomarkers and adverse pregnancy outcomes among GH subjects (*p* > 0.05).

**Table 5:** Spearman rho correlation of Angiogenic and Oxidative biomarkers with antepartum and postpartum adverse pregnancy outcome in Gestational hypertensive women

	PLGF	sFlt-1	8-epi-PGF2α	T-AOC
Antepartum complications				
IUGR	*r* = −0.109;	*r* = 0.027;	*r* = 0.009;	*r* = −0.203;
	*p* = 0.060	*p* = 0.901	*p* = 0.725	*p* = 0.197
Placental abruptio/praevia	*r* = −0.033;	*r* = 0.040;	*r* = 0.022;	*r* = −0.107;
	*p* = 0.683	*p* = 0.259	*p* = 0.901	*p* = 0.443
IUFD	*r* = −0.191;	*r* = 0.150;	*r* = 0.083;	*r* = −0.196;
	*p* = 0.071	*p* = 0.092	*p* = 0.724	*p* = 0.173
Postpartum adverse outcomes				
Stillbirth	*r* = −0.066;	*r* = 0.001;	*r* = 0.113;	*r* = −0.191;
	*p* = 0.483	*p* = 0.933	*p* = 0.270	*p* = 0.420
PPH	*r* = −0.057;	*r* = 0.019;	*r* = 0.181;	*r* = −0.159;
	*p* = 0.183	*p* = 0.396	*p* = 0.317	*p* = 0.158

r: correlation coefficient; *p* < 0.05 (statistically significant) *p* < 0.001 (statistically highly significant) *p* < 0.0001 (statistically very highly significant). *r* < 0.5 (weak correlation); *r* > 0.5 (strong correlation)

## Discussion

Hypertension in pregnancy especially preeclampsia has been linked with maternal mortality, premature birth, IUGR, stillbirth and other adverse outcomes [3, 7]. However, the mechanism linking adverse pregnancy outcomes and preeclampsia is unknown. This study assessed the correlation between angiogenic regulators and oxidation stress markers with adverse pregnancy outcomes among Ghanaian preeclamptic and gestational hypertensive women. Imbalance in angiogenic regulators was identified by an increased in sFlt-1 with a corresponding decreased in PLGF levels among PE subjects with complications. Oxidative stress was observed amongst the participants with GH, PE and PE co-existing with adverse pregnancy outcomes as depicted by the high lipid peroxidation and reduced TAOC levels. Furthermore, angiogenic and oxidative stress biomarkers correlated significantly with IUGR, IUFD, placental abruptio, stillbirth and PPH. The findings of this study has demonstrated that women with preeclampsia complicated by IUGR had a markedly widespread endothelial dysfunction depicted by the extremely elevated sFlt-1 and reduced PLGF levels compared to normal pregnancy and PE without associated complications (Fig. 2). A previous study by Ghosh et al. [19] observed reduced levels of PLGF in PE but not others [11]. Imbalance in angiogenic regulators leading to placental bed hypoxia and a subsequent endothelial dysfunction may have ultimately resulted in IUGR [19]. Our finding shows that preeclamptic pregnancy complicated by IUGR is one of the probable causes of low birth weight (LBW) infants (Fig. 3). Again the findings of this study shows a significantly elevated sFlt-1 and a corresponding decreased PLGF levels observed in PE women with IUFD, placental previa, placental abruptio and HELLP syndrome compared to those with PE only have not been reported by previous studies. The mechanism of these finding though not well understood, an overall reduction of foetal perfusion of the placenta and shallow trophoblasts invasion have been implicated [19]. The present study indicates that angiogenic growth factors may be reliable biomarkers for the prediction of adverse pregnancy outcomes (IUGR, IUFD, placental abruptio, stillbirth, and PPH). Biochemical imbalance in preeclampsia may be associated with an increase oxidative stress depicted by increased lipid peroxidation as well as a defective antioxidant protection. In addition to the defective angiogenic regulators, elevated 8-epiPGF2α and reduced TAOC were significantly linked with IUGR, IUFD, placental previa, placental abruptio and HELLP syndrome pregnancies in this present study. PE associated with adverse outcomes may have created disequilibria in the levels of pro-oxidants (increased 8-epiPGF2α) and antioxidants proportionate to the degree of severity (reduced TAOC) culminating in oxidative stress. Significantly high lipid peroxidation and a compromised antioxidant system emphasizes that oxidative stress play a pivotal role in the etiology of adverse pregnancy outcome. Some studies [11, 19] indicated that proangiogenic and anti-angiogenic proteins may be useful biomarkers in predicting pregnancy complications such as pre-eclampsia and fetal growth restriction. Ghosh et al. [19] have reported a significant negative correlation between PlGF and IUGR which is consistent with the present study (Table 4). This study is the first to observe a significant negative correlation between PLGF and IUFD, placental abruptio, stillbirth, and PPH and a positive correlation between sFlt-1 and IUGR, IUFD, placental abruptio, stillbirth, and PPH. This probably indicates that the degree of sFlt-1 is elevated while PLGF is reduced proportionately to the hypertensive pregnancy complicated with adverse outcomes. The markedly increased sFlt-1 and corresponding decreased PLGF levels proportional to the severity of PE condition indicates that imbalances in angiogenic regulators play an important role in the etiology of these adverse outcomes. Hypoxia induced by incomplete trophoblasts invasion and placental underperfusion may explain these findings [19]. The present study has established a relationship between oxidative stress as indicated by high 8-epiPGF2α and low TAOC with adverse pregnancy outcome (Table 4). As 8-epiPGF2α correlated positively with adverse pregnancy outcome (IUGR, IUFD, placental abruptio, stillbirth, and PPH) in preeclamptic participants, the correlation of TAOC with IUGR, IUFD, placental abruptio, stillbirth, and PPH levels was significant and inversely correlated. However, no significant correlation between adverse pregnancy outcome and markers of angiogenic factors and oxidative stress biomarkers were observed among women presenting with GH in this study (Table 5).

PE associated adverse outcome may be using up the antioxidant proteins in circulation for its metabolic processes and this could explain the reduced concentration of TAOC. Adverse pregnancy outcome were mostly associated with preeclamptic pregnancies than gestational hypertension indicating that pregnancies complicated by high blood pressure and proteinuria stands an increased risk of suffering these adverse outcomes than normal pregnancy and GH. The increase in sFlt-1 and 8-epiPGF2α in PE is an indication that these markers may play a synergistic role in the pathogenesis of PE and may be responsible for the widespread endothelial dysfunction culminating in these adverse pregnancy outcomes. The limitation of this study was the small sample size used. However, the imbalance in the levels of angiogenic factors and oxidative stress biomarkers corresponded well with some previous studies. This study will serve as a baseline and thus further studies with prospective cohort design involving large sample size will be needed to establish the strength of this observation.

## Conclusion

PE is associated with an imbalance in the angiogenic regulators (increased sFlt-1 and decreased PLGF) and oxidative markers (increased 8-epiPGF2α and decreased TAOC) proportionate to the adverse pregnancy outcomes. Early identification of these imbalance would alert health care givers in anticipation of a possible adverse pregnancy outcome and thus increased surveillance during pregnancy and parturition and put in the necessary preventive measures to ameliorate the adverse outcome.

## Abbreviations

GH: Gestational hypertension
PE: Preeclampsia
PLGF: Placental growth factor (PlGF)
sFlt-1: Soluble fms-like tyrosine kinase 1
8-epi-PGF2α: 8-epi-prostaglandin F2alpha
T-AOC: Total antioxidant capacity
ROS: Reactive oxygen species
IUGR: Intrauterine growth restriction
IUFD: Intrauterine fetal death
PPH: Postpartum haemorrhage
PPROM: Premature prerupture of membranes
NP: Normal pregnancy
